# Numerical model of protein crystal growth in a diffusive field such as the microgravity environment

**DOI:** 10.1107/S0909049513022784

**Published:** 2013-10-01

**Authors:** Hiroaki Tanaka, Susumu Sasaki, Sachiko Takahashi, Koji Inaka, Yoshio Wada, Mitsugu Yamada, Kazunori Ohta, Hiroshi Miyoshi, Tomoyuki Kobayashi, Shigeki Kamigaichi

**Affiliations:** a Confocal Science Inc., Hayakawa 2nd Building 7F, 2-12-2 Iwamoto-cho, Chiyoda-ku, Tokyo 101-0032, Japan; b Neo Force, 5-9-14-403 Tsurumaki, Setagaya-ku, Tokyo 154-0016, Japan; c Maruwa Foods and Biosciences Inc., 170-1 Tsutsui-cho, Yamatokoriyama, Nara 639-1123, Japan; d Japan Aerospace Exploration Agency, 2-1-1 Sengen, Tsukuba, Ibaraki 305-8505, Japan

**Keywords:** microgravity, transient and homogeneous field, transient and diffusive field, protein crystal, numerical model, protein depletion zone, impurity depletion zone

## Abstract

It is said that the microgravity environment positively affects the quality of protein crystal growth. The formation of a protein depletion zone and an impurity depletion zone due to the suppression of convection flow were thought to be the major reasons. In microgravity, the incorporation of molecules into a crystal largely depends on diffusive transport, so the incorporated molecules will be allocated in an orderly manner and the impurity uptake will be suppressed, resulting in highly ordered crystals. Previously, these effects were numerically studied in a steady state using a simplified model and it was determined that the combination of the diffusion coefficient of the protein molecule (*D*) and the kinetic constant for the protein molecule (β) could be used as an index of the extent of these depletion zones. In this report, numerical analysis of these depletion zones around a growing crystal in a non-steady (*i.e.* transient) state is introduced, suggesting that this model may be used for the quantitative analysis of these depletion zones in the microgravity environment.

## Abstract 2

Numerical analysis of the concentration depletion zones in a transient state suggested that, in microgravity, protein crystals grow in a lower supersaturation and the impurity ratio decreases in the centre of the crystal.

## 1. Introduction

Protein crystal growth experiments are a promising area in the usage of microgravity to contribute to structural biology (McPherson, 1999 ▶; Littke & John, 1986 ▶; Kundrot *et al.*, 2001 ▶; Vergara *et al.*, 2003 ▶). When protein molecules are taken into a crystal, a spherical area of low protein concentration is formed around the growing crystal. In the terrestrial environment, a density-driven flow occurs to supply protein molecules to the low concentration area, disturbing this area. However, in microgravity, this density-driven flow does not occur, so the protein molecules are supplied to the crystal only by thermal diffusion caused by Brownian motion. Therefore, the low protein concentration area around the growing crystal is maintained, resulting in the formation of a protein concentration depletion zone (protein depletion zone, PDZ). The PDZ helps grow the crystal at a low supersaturation (Chernov, 1998 ▶; Otálora *et al.*, 2001 ▶), eventually suppressing the disorder of protein molecules in the crystal. Following similar steps as the protein molecules, a low-impurity concentration area around the growing crystal (impurity depletion zone, IDZ) is formed (Chernov, 1998 ▶; Thomas *et al.*, 2000 ▶), also suppressing crystal disorder.

The PDZ and IDZ in microgravity in the steady and diffusive states have been analyzed and discussed (Tanaka *et al.*, 2004 ▶). A steady state occurs when there is a constant concentration of the protein in a solution far from a growing crystal. The diffusive state occurs when the molecules diffuse in a convection-free environment. We have compared the extent of PDZ and IDZ formation in steady and diffusive states with the formations in steady and homogeneous states and discussed how the PDZ and IDZ affected protein crystal growth in microgravity (Tanaka *et al.*, 2004 ▶; Inaka *et al.*, 2012 ▶).

However, in reality, the process of crystal growth in a conventional protein crystal growth experiment occurs in a non-steady transient state, decreasing protein concentration in the solution as the protein molecules are incorporated into the crystal. Therefore, it would be valuable to have the ability to employ a mathematical model corresponding to the transient state to know under what conditions the crystals really grow and how much impurity is taken into the crystals, quantitatively.

Here we introduce a rather simple numerical calculation model for expressing the transient state. We can compare the transient and homogeneous state (terrestrial gravity) with the transient and diffusive state (microgravity), and propose that this model can be applied to the examination of the process of protein crystallization both in microgravity and terrestrial environments, quantitatively.

## 2. Calculation model

### 2.1. Model of the field around a growing crystal

**Figure 1:**
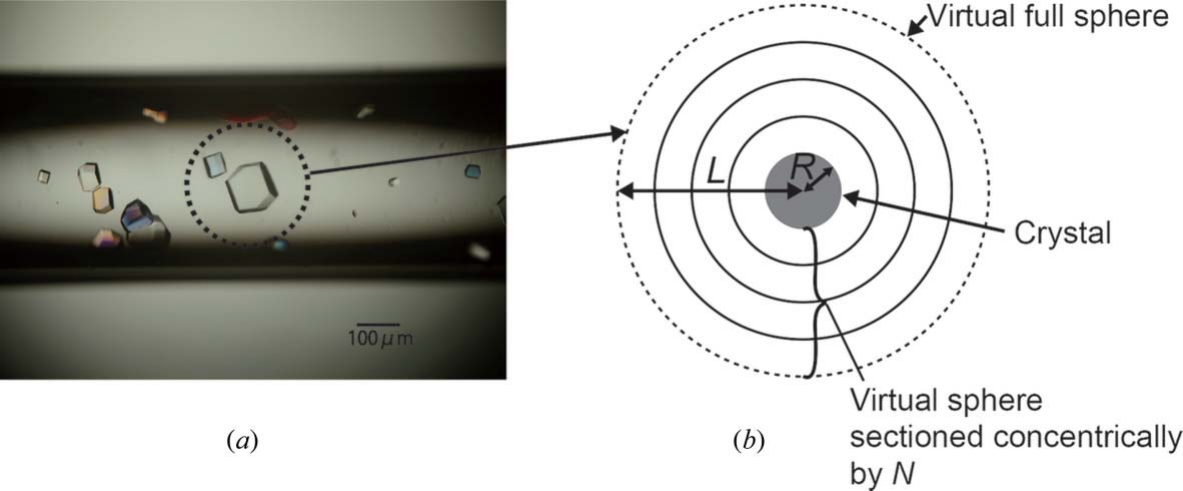
The conceptual configuration of the numerical model for crystal growth. (*a*) In actual crystallization, several crystals are grown in a solution. (*b*) To simplify this process for the model, a crystal is assumed to grow spherically in a virtual sphere, of which the radius, *L*, is related to the amount of the protein uptake into the crystal [see equation (1)]. To accommodate the diffusive process, the area between the surface of the crystal and the virtual sphere are sectioned concentrically by *N*. The diffusive processes are considered to occur between the inner section and outer section.

There are many kinds of crystallization methods such as the vapour-diffusion method, dialysis, and the counter-diffusion method, *etc*. In our calculation model, for simplicity, we assume the batch method is used to crystallize protein because in this method the precipitant concentration and protein solubility do not change from the beginning to the end of crystallization. As for the nucleation, we assume that all nuclei start growing simultaneously and the final size of the crystal is the same for all crystals. In this model, we assume that crystallization occurs in a virtual sphere of radius *L* as shown in Fig. 1 ▶ and that the shape of the crystal is a sphere with a radius of *R* to simplify the calculation.

When the final size of the crystal is *R*(∞),
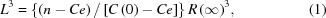
where *C*(0) and *Ce* are the protein concentration at the beginning and at the end (solubility) of crystallization, respectively, and *n* is the weight density of the crystal.

The velocity of crystal growth is (Chernov, 1998 ▶)
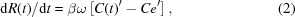
where *C*(*t*)′ is the number of protein molecules in a unit of volume (1/cm^3^) at time *t* after crystallization has occurred, *Ce*′ is the number of protein molecules in a unit of volume at the protein solubility concentration and β is the kinetic constant of a protein molecule. ω is the volume for one molecule and can be defined as ω = *M*/(*n* *N*_A_), where *M* is the protein molecular weight and *N*_A_ is Avogadro’s number.

For experimental purposes, we usually express concentration as weight per volume at time *t*, *C*(*t*). Therefore, *C*(*t*)′ is expressed as 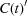 = 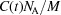; (2) is replaced with (Tanaka *et al.*, 2004 ▶)
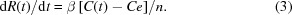
Therefore, the weight of protein attaching to a crystal surface in an iota of time is
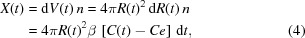
where *V*(*t*) and *R*(*t*) are the volume and the radius of crystal at time *t*, respectively.

When an impurity contaminates the solution, and if the impurity attaches to the surface of the crystal at a fixed ratio and the reverse reaction is ignored, the weight of the impurity attaching to the crystal surface in an iota of time is
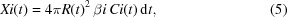
where β*i* is the kinetic constant of the impurity molecule and *Ci*(*t*) is the impurity concentration on the surface of the crystal at time *t*.

### 2.2. Transient and homogeneous model and transient and diffusive model

In the transient and homogeneous model (THM) which represents crystal growth in the terrestrial environment, the concentration of the protein in the solution around the growing crystal is uniform and the sum of the total amount of the protein in the solution and in the crystal is constant. Therefore the concentration of the protein solution during crystal growth can be expressed as
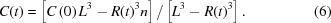
Substituting (6) in (3),
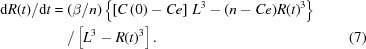
Therefore, the crystal radius can be obtained from the re­peated calculation of (7) using the difference equation (Tanaka *et al.*, 2004 ▶),
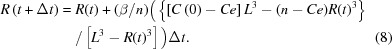
Considering the total amount of the impurity in the solution and in the crystal is constant, the concentration of the impurity in the solution can be obtained by the following equation,
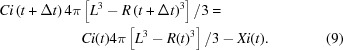
Substituting (5) into (9), the impurity concentration in the solution can be obtained from the repeated calculation of the difference equation below,
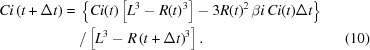
On the other hand, in the transient and diffusive model (TDM) which represents the microgravity environment, we have to consider both the diffusion process in the virtual sphere of radius *L* and the crystal growth process in the centre of the virtual sphere. The three-dimensional diffusion equation is 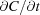 = 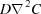, but in the case of spherical coordinates the partial differential equation is
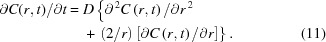
This can be applied to the area between the surface of the crystal and the virtual sphere of radius *L*. For the outer boundary where *r* is equivalent to *L*, no-flux of the material is assumed. For the boundary conditions on the surface of the crystal, the process of the crystal growth is
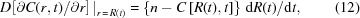
where *C*(*r*, *t*) is the protein concentration at the position of *r* from the centre of the virtual sphere at time *t*. The diffusion equation for impurities is the same as that for protein, as shown below with similar boundary conditions,
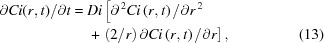
and the process of the impurity uptake into the crystal is
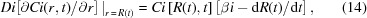
where *Ci*(*r*, *t*) is the impurity concentration at the position of *r* from the centre of the virtual sphere at time *t*.

To solve these partial differential equations numerically, it is common to divide a sphere along a radius into the same intervals to apply difference equations. However, in the case of a growing crystal, the sections are adsorbed into the crystal one after another. We may include a conditional judgment on whether the sections are embedded in the crystal into the partial differential equation. It has been found that these calculations may result in intolerant errors caused by the discontinuity of the protein concentration on the surface of the crystal when the section is incorporated into the crystal.

Therefore, we divide the sphere into *N* sections with a variable length, Δ*r*(*t*), from the surface of the crystal to the surface of the virtual sphere whose radius is *L* and examine the diffusion in those sections. If the crystal radius is *R*(*t*) at time *t*,
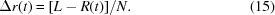
The section number *i* (*i* = 1, 2, 3,…, *N*) is placed between two spheres whose radii are 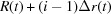 and 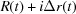.

The process of the calculation for one time step of the repeated calculation of the difference equation is the following:

(i) Calculate the crystal growth: based on (3), we can calculate the increase of the crystal radius by the following equation.
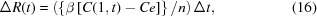
where 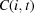 is the concentration of the protein in the *i*th section at time *t*; and the radius after one time unit Δ*t* is
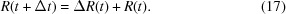



(ii) Calculate the protein concentration of the first section which contacts with the growing crystal: considering (12), the protein concentration of this section is affected by its adsorption onto the crystal surface and by the diffusive mass transfer from the next section. For the first process, the following relation is conserved, since the amount of protein which is adsorbed on the crystal is equal to the decreased amount of it from the section,
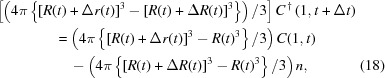
where 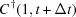 is the concentration of the protein in the first section after time 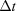 with the protein adsorption occurring in the first section.

For the second process, the following difference equation is derived from (11) neglecting the diffusion from the inner section,
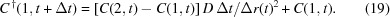
Therefore, combining these two processes, the protein concentration of the first section can be expressed as
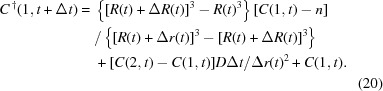



(iii) Calculate the diffusive protein transfer between the surface of the crystal to the surface (*i *= 2 to *N*) of the virtual sphere: the difference equation for this process is derived from (11),
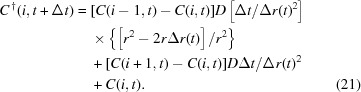



(iv) Calculate the impurity concentration of the first section which contacts with the growing crystal: considering (14), the protein concentration of this section is affected by its adsorption onto the crystal and by the diffusive mass transfer from the next section. For the first process, the following relation is conserved, since the amount of the impurity which is adsorbed on the crystal is equal to the decrease of it from the section,
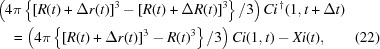
where 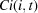 is the concentration of the impurity in the *i*th section at time *t*, and 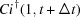 is the concentration of the impurity in the first section after one time unit Δ*t*.

For the second process, the following difference equation is derived from (13) neglecting the diffusion from the inner section,
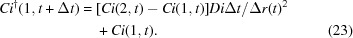
Therefore, by combining these two processes the impurity concentration of the first section can be expressed as
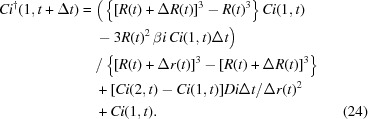



(v) Calculate the diffusive impurity transfer between the surface of the crystal to the surface of the virtual sphere: the difference equation for this process is derived from (13),
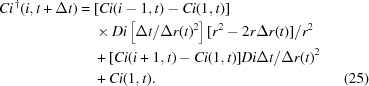



(vi) Re-sectioning the sphere: after the crystal has grown during one time unit Δ*t*, each section moves slightly to the outer position. Therefore, we re-divide the sphere into *N* sections with variable length 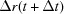,
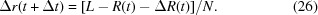
The concentration of the new section, whose number is *i*, is expressed as 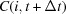, and can be calculated by considering the conservation of the amounts of the protein and the impurity,
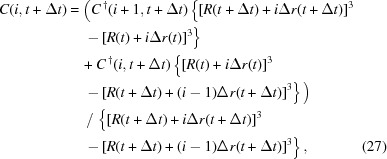


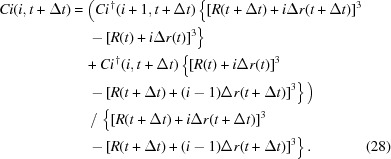
The calculation program was created in Microsoft C++ and was executed in Windows 7 or Windows XP. *N* was defined as 240 and Δ*t* as 2 × 10^−5^ (h). The repeated calculation for *t* = 1000 (h) took about 1500 s with a conventional desktop PC. After the calculation was finished, the calculated final crystal size was almost the same as that we actually obtained (±0.02%). Therefore, we concluded that the program ran with satisfactory precision for the comparison of crystal growth in space and on the ground.

## 3. Lysozyme crystal growth in THM and TDM

### 3.1. Parameter calculations based on experiment results

To use some realistic parameters, we referenced lysozyme crystallization using the batch method. Purified lysozyme (20 mg ml^−1^) was crystallized using 0.7 *M* sodium chloride as a precipitant in 50 m*M* sodium acetate pH 4.5. The final size of the crystal [*R*(∞)] and the final protein concentration in the solution (*Ce*) were measured, and the kinetic constant of the protein molecule (β) and the diffusion coefficient of the protein molecule (*D*) were estimated as shown in Table 1 ▶ (Tanaka *et al.*, 2012 ▶).

### 3.2. Lysozyme crystal growth in THM and TDM

**Figure 2:**
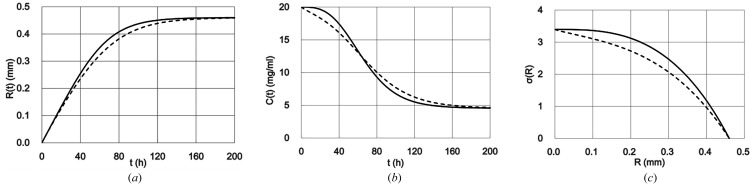
Calculated results of the lysozyme crystal growth in the salt condition in the homogeneous (solid line) and diffusive (dotted line) model in the transient state. (*a*) Time course of the crystal growth. (*b*) Time course of the protein concentration on the crystal surface. (*c*) Supersaturation level around the growing crystal. *R*(*t*), *R*: radius of the crystal (mm); *C*(*t*): concentration of the protein on the surface of the crystal (mg ml^−1^); σ(*R*): supersaturation level; *t*: time after the crystal starts growing.

The time course of lysozyme crystal growth is shown in Fig. 2(*a*) ▶. The solid line is for THM obtained from the repetition of calculation (8) and the dotted line is for TDM obtained from the repetition of calculation of equations (16), (17), (20), (21), (26) and (27). This figure indicated that the crystal growth in TDM is a little slower than in THM. This might suggest that, in the case of THM, the fast uptake of the protein molecules into the crystal was accelerated by the transportation of protein molecules toward the crystal by convection flow. However, in the case of TDM, the PDZ deprived the protein molecules from around the growing crystal and only the diffusive flow could deliver protein molecules to the crystal, so it kept the protein concentration around the surface of the crystal lower than in THM and caused slower crystal growth.

### 3.3. Average protein supersaturation level

The degree of supersaturation on the surface of the crystal while it is growing is defined as
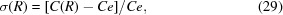
where *C*(*R*) is the concentration of the protein on the surface of the crystal when the crystal radius is *R*. It is said that the quality of the crystal, indexed by the X-ray diffraction resolution, mosaicity and/or *R*_merge_, is better if it grows at the lower supersaturation (McPherson, 1999 ▶) although dependency of those on σ(*R*) has not been verified yet. To know the supersaturation level on the surface of the crystal while it is growing, the time course of the protein concentration on the crystal surface was calculated as shown in Fig. 2(*b*) ▶. The solid line is for THM obtained from equation (6) and the dotted line is for TDM obtained from equation (27). Figs. 2(*a*) ▶ and 2(*b*) ▶ were combined to create Fig. 2(*c*) ▶ using equation (29). The solid line and dotted line are for THM and TDM, respectively. It was found that the supersaturation level was high in the centre of the crystal and gradually became lower toward the surface of the crystal. Over the full range of crystal growth, the supersaturation level was slightly lower in TDM than in THM. At the end of crystal growth, the supersaturation level was the same as the protein solubility both in THM and TDM.

Since X-ray diffraction intensity depends on the volume of the protein molecules in a crystal (McPherson, 1999 ▶), the quality of an X-ray diffraction image may depend on the integrated diffraction images obtained from a certain volume of the crystal which was grown in changeable supersaturation. Therefore, as a quantitative index of supersaturation, the average supersaturation level (ASS) was defined as the integrated σ as the crystal grew, averaged by the total volume,
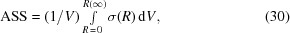
where σ(*R*) is the degree of supersaturation at the surface of the crystal when the crystal radius is *R*.

**Figure 3:**
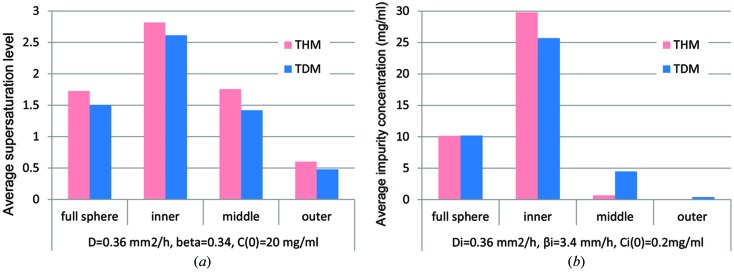
(*a*) Average supersaturation level, and (*b*) average impurity concentration in each section for crystallization of purified lysozyme.

To compare the ASS within one crystal, the crystal was divided into three sections by volume from the centre of the crystal. The radius of the inner third of the crystal is 69.3% of *R*(∞), the middle 87.4%, and the outer 100%. ASS in THM and TDM are shown in Fig. 3(*a*) ▶. The ASS for a full sphere in THM was 1.73 and in TDM was 1.50 which was about 87% of that of the THM. In each third of the crystal volume, TDM was lower than THM. These findings are consistent with the estimations obtained by former analyses using the steady state model (Tanaka *et al.*, 2004 ▶; Inaka *et al.*, 2012 ▶).

### 3.4. Average impurity concentration

As for impurity, it is not realistic to qualify and quantify the impurity molecules in a protein solution. Therefore, we assumed a contaminating impurity, whose initial concentration, *Ci*(0), was 1% of that of the protein, *C*(0), with a diffusion coefficient, *Di*, the same (0.36 mm^2^ h^−1^) as that of the protein, *D,* and a kinetic constant, β*i,* ten times (3.4 mm h^−1^) as large as that of the protein β. *Di* was equal to *D* because we assumed that the molecular weight of the impurity and the protein were the same. A β*i* ten times larger meant that the impurity molecule had a ten times higher affinity to the crystal than the protein molecule. Actually, when acetylated lysozyme was an impurity, it was reported that the impurity attached to the crystal with an affinity several multiples of ten times higher than the lysozyme protein (Thomas & Chernov, 2001 ▶).

**Figure 4:**
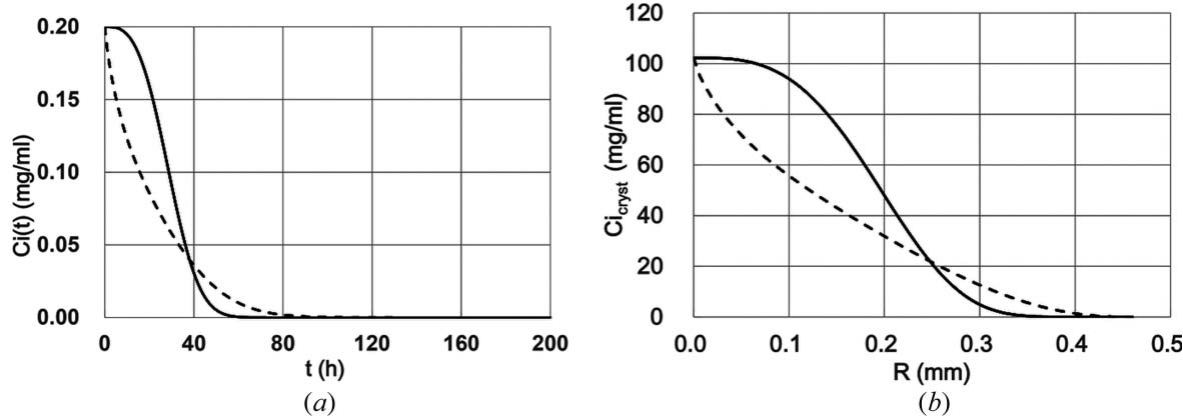
Calculation results of the impurity concentration in salt conditions in the homogeneous (solid) and diffusive (dotted) model in the transient state. (*a*) Time course of the impurity concentration on the crystal surface. (*b*) Impurity concentration around the growing crystal. *R*: radius of the crystal (mm); *Ci*(*t*): concentration of the impurity on the surface of the crystal (mg ml^−1^); *Ci*_cryst_(*R*): concentration of the impurity in the crystal at *R* from the centre of the crystal; *t*: time after the crystal starts growing.

The time course of the concentration of the impurity around the surface of the crystal is shown in Fig. 4(*a*) ▶. The solid line is for THM obtained from equation (10) and the dotted line is for TDM from equations (16), (17), (24), (25), (26) and (28).

The concentration of the impurity attached to the crystal with a radius *R* from the centre of the crystal [*Ci*_cryst_(*R*)] can be described as
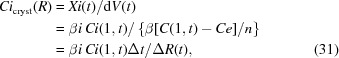
where *Xi*(*t*) is the weight of the impurity attached to the crystal surface in a unit of time, and *C*(*t*) and *Ci*(*t*) are concentrations of the protein and the impurity on the surface of the crystal at time *t*. Generally, if many impurity molecules attach to a crystal, a highly disordered crystal may grow. Therefore, to determine the impurity concentration inside the crystal, Figs. 2(*a*) ▶ and 4(*a*) ▶ were combined and equation (30) was applied to create Fig. 4(*b*) ▶. The solid and dotted lines are for THM and TDM, respectively. As shown in Fig. 4(*b*) ▶, if the radius of the crystal was between 0 and 0.25 mm, the concentration of the impurity on the surface of the crystal in TDM was much lower than that of THM. Then, if the radius was larger than 0.25 mm, the impurity concentration in TDM became higher than that of THM, but much lower than that at the beginning of the crystal growth, and the concentration of impurity around the crystal became zero at the end of crystal growth.

Similar to the ASS, the average impurity concentration (AIC) was defined as
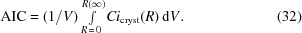
As in the ASS, the AIC was calculated for a full sphere crystal and in each of the three sections as in Fig. 3(*b*) ▶. The AIC of TDM and THM were the same for a full sphere crystal because all of the impurity was finally adsorbed into the crystal. In Fig. 3(*b*) ▶, the AIC was high in the inner third of the crystal and fell as the crystal grew in both TDM and THM. In the inner third of the crystal the AIC was lower in TDM than in THM, but in the middle section of the crystal the AIC was higher in TDM than in THM. In the outer section of the crystal, the impurity concentration was almost zero in THM and TDM. These might suggest that, in TDM, the IDZ was formed around the crystal in the diffusive field and deprived the impurity molecules around the crystal surface, but, in THM, the fast attachment of the impurity molecules to the crystal lowered the concentration of the impurity more quickly. These findings could not be elucidated by the steady state model performed previously.

## 4. Conclusion

From the results of the model calculation, it became clear quantitatively that all the sections of the crystal grown in THM and TDM are surrounded by different supersaturation levels of protein and different concentrations of impurity when they grow. Therefore, for X-ray diffraction experiments, we recommend considering how the quality difference within a whole crystal, caused by the difference in protein supersaturation and impurity concentration levels during crystal growth, may affect the quality of the X-ray diffraction patterns.

As for protein supersaturation, it became clear that, in a diffusive model such as in microgravity, protein crystals grow in a lower supersaturation level of protein than in a homogeneous field. This may be due to the effects of the PDZ.

Regarding impurity, its concentration is higher in THM than in TDM when the inner section of the crystal grows. In the middle section, although the impurity concentration is higher in TDM than in THM, it is much lower than in the inner section both in THM and TDM. This may be due to the effects of IDZ and the faster uptake of the impurity molecules into the crystal.

It was reported that the growth of the middle and outer sections of a crystal are influenced by the molecular order of the inner section of the crystal as shown with X-ray tomography (Sawaura *et al.*, 2011 ▶). Therefore, we speculate that the outer section of crystals grown in TDM may have better quality because the supersaturation ratios and impurity concentrations are lower in the inner section of the crystals grown in TDM than those of the crystals grown in THM. This may explain the reason why some better quality crystals grew in microgravity (Tanaka *et al.*, 2007 ▶, 2011 ▶; Takahashi *et al.*, 2010 ▶). Further examination is required to know the influence of the ASS and AIC on the X-ray diffraction patterns quantitatively.

In this study, we have fixed β as a constant. Actually the increase of the impurity concentration reduces the velocity of the crystal growth (Nakada *et al.*, 1999 ▶). Thus, for the next step, we will proceed to analyze numerically how the diffusive model of protein crystal growth is affected by the increase of the initial amount of the impurity.

Here we have introduced a practical mathematical model which can be calculated with an ordinary personal computer. Using this transient model, further numerical analyses of higher viscosity of the crystallization solution (lower *D*) and of higher homogeneity of the protein sample (higher β) will be examined for the verification of the effectiveness of these values on the improvement of crystal quality in microgravity; and, in the near future, we will use this model on the results obtained in the JAXA PCG crystallization experiments to further understand the effects of microgravity.

## Figures and Tables

**Table 1:** Initial parameters for the calculation of the homogeneous and the diffusive models

Crystallization condition	*R*(∞) (mm)†	*Ce* (mg ml^−1^)‡	β (mm h^−1^)	*D* (mm^2^ h^−1^)	*n* (g cm^−3^)
Salt§	0.46	4.55	0.34	0.360	0.79

† Average of the final size of the crystal radius.

‡ Average of the final protein concentration.

§ 20 mg ml^−1^ purified lysozyme, 0.7 *M* sodium chloride, 50 m*M* sodium acetate pH 4.5.

## References

[bb1] Chernov, A. A. (1998). *Acta Cryst.* A**54**, 859–872. 10.1107/s0108767398008587 9859196

[bb2] Inaka, K., Tanaka, H., Takahashi, S., Sano, S., Sato, M., Shirakawa, M. & Yoshimura, Y. (2012). *Defect Diffus. Forum*, **323**–**325**, 565–569.

[bb3] Kundrot, C. E., Judge, R. A., Pusey, M. L. & Snell, E. H. (2001). *Cryst. Growth Des.* **1**, 87–99.

[bb4] Littke, W. & John, C. (1986). *J. Cryst. Growth*, **76**, 663–672.

[bb5] McPherson, A. (1999). *Crystallization of Biological Macromolecules.* New York: Cold Spring Harbor Laboratory Press.

[bb6] Nakada, T., Sazaki, G., Miyashita, S., Durbin, S. D. & Komatsu, H. (1999). *J. Cryst. Growth*, **196**, 503–510.

[bb7] Otálora, F., Novella, M. L., Gavira, J. A., Thomas, B. R. & García Ruiz, J. M. (2001). *Acta Cryst.* D**57**, 412–417. 10.1107/s0907444901000555 11223518

[bb8] Sawaura, T., Fujii, D., Shen, M., Yamamoto, Y., Wako, K., Kojima, K. & Tachibana, M. (2011). *J. Cryst. Growth*, **318**, 1071–1074.

[bb9] Takahashi, S., Tsurumura, T., Aritake, K., Furubayashi, N., Sato, M., Yamanaka, M., Hirota, E., Sano, S., Kobayashi, T., Tanaka, T., Inaka, K., Tanaka, H. & Urade, Y. (2010). *Acta Cryst.* F**66**, 846–850. 10.1107/S1744309110020828 PMC2898477 20606289

[bb10] Tanaka, H., Inaka, K., Furubayashi, N., Yamanaka, M., Takahashi, S., Sano, S., Sato, M., Shirakawa, M. & Yoshimura, Y. (2012). *Defect Diffus. Forum*, **323**–**325**, 549–554.

[bb11] Tanaka, H., Inaka, K., Sugiyama, S., Takahashi, S., Sano, S., Sato, M. & Yoshitomi, S. (2004). *Ann. NY Acad. Sci.* **1027**, 10–19. 10.1196/annals.1324.002 15644341

[bb12] Tanaka, H., Tsurumura, T., Aritake, K., Furubayashi, N., Takahashi, S., Yamanaka, M., Hirota, E., Sano, S., Sato, M., Kobayashi, T., Tanaka, T., Inaka, K. & Urade, Y. (2011). *J. Synchrotron Rad.* **18**, 88–91. 10.1107/S0909049510037076 PMC3004263 21169700

[bb13] Tanaka, H., Umehara, T., Inaka, K., Takahashi, S., Shibata, R., Bessho, Y., Sato, M., Sugiyama, S., Fusatomi, E., Terada, T., Shirouzu, M., Sano, S., Motohara, M., Kobayashi, T., Tanaka, T., Tanaka, A. & Yokoyama, S. (2007). *Acta Cryst.* F**63**, 69–73. 10.1107/S1744309106054625 PMC2330117 17277442

[bb14] Thomas, B. R. & Chernov, A. A. (2001). *J. Cryst. Growth*, **232**, 237–243.

[bb15] Thomas, B. R., Chernov, A. A., Vekilov, P. G. & Carter, D. C. (2000). *J. Cryst. Growth*, **211**, 149–156.

[bb16] Vergara, A., Lorber, B., Zagari, A. & Giegé, R. (2003). *Acta Cryst.* D**59**, 2–15. 10.1107/s0907444902021443 12499533

